# Transgenic rice plants expressing synthetic *cry2AX1* gene exhibits resistance to rice leaffolder (*Cnaphalocrosis medinalis*)

**DOI:** 10.1007/s13205-015-0315-4

**Published:** 2016-01-05

**Authors:** R. Manikandan, N. Balakrishnan, D. Sudhakar, V. Udayasuriyan

**Affiliations:** Department of Plant Biotechnology, Centre for Plant Molecular Biology and Biotechnology, Tamil Nadu Agricultural University, Coimbatore, 641 003 India

**Keywords:** Cry2AX1, Transgenic rice, Insect resistance, Rice leaffolder

## Abstract

*Bacillus thuringiensis* is a major source of insecticidal genes imparting insect resistance in transgenic plants. Level of expression of transgenes in transgenic plants is important to achieve desirable level of resistance against target insects. In order to achieve desirable level of expression, rice chloroplast transit peptide sequence was fused with synthetic *cry2AX1* gene to target its protein in chloroplasts. Sixteen PCR positive lines of rice were generated by *Agrobacterium* mediated transformation using immature embryos. Southern blot hybridization analysis of T_0_ transgenic plants confirmed the integration of *cry2AX1* gene in two to five locations of rice genome and ELISA demonstrated its expression. Concentration of Cry2AX1 in transgenic rice events ranged 5.0–120 ng/g of fresh leaf tissue. Insect bioassay of T_0_ transgenic rice plants against neonate larvae of rice leaffolder showed larval mortality ranging between 20 and 80 % in comparison to control plant. Stable inheritance and expression of *cry2AX1* gene was demonstrated in T_1_ progenies through Southern and ELISA. In T_1_ progenies, the highest concentration of Cry2AX1 and mortality of rice leaffolder larvae were recorded as 150 ng/g of fresh leaf tissue and 80 %, respectively. The Cry2AX1 expression even at a very low concentration (120–150 ng/g) in transgenic rice plants was found effective against rice leaffolder larvae.

**Electronic supplementary material:**

The online version of this article (doi:10.1007/s13205-015-0315-4) contains supplementary material, which is available to authorized users.

## Introduction

Globally, more than 3 billion people depend on rice (*Oryza sativa*, L.) as their staple food, and by 2050 about 30 % more rice must be produced to meet the needs of the growing population (Brookes and Barfoot 2003). One of the major constraints in rice production, throughout the rice growing countries of the world, is the menace of insect pests. Among them the lepidopteran insects, such as yellow stem borer (YSB) (*Scirphophaga incertulus* Walker), striped stem borer (SSB) (*Chilo supressalis* Walker) and rice leaffolder (RLF) (*Cnaphalocrosis medinalis* Guenee*)* are the major ones, causing significant yield losses up to 10 and 30 % on an average, respectively, each year (Krishnaiah and Varma 2012). The uses of chemical insecticides against the boring insects have not found much effective as the insecticides cannot reach them (Deka and Barthakur 2010). Chemical control besides increasing the cost of production also causes deleterious effects to the ecosystem. The development of insect-resistant lines in rice through conventional breeding has been a challenge due to non-availability of resistance source in germplasm collection (Bhattacharya et al. 2006). Thus, an alternative and the most attractive strategy is to employ the tools of genetic engineering to produce insecticidal proteins by introducing heterologus insecticidal genes.

Insecticidal crystal protein gene (*cry* gene) of *Bacillus thuringiensis* (Bt) is effective against important crop pests and widely used in plant genetic engineering. Bt mediated insect-resistant crop technology is the most successful application of agricultural biotechnology in today’s agriculture. Several crops such as tomato, cotton, maize and rice have been successfully transformed with different versions of crystal protein gene of Bt (Mandaokar et al. 2000; Sakthi et al. 2015; Jansen et al. 1997; Chen et al. 2005). The first transgenic rice plant with insect-resistant Bt protein (Cry1Ab) was reported by Fujimoto et al. (1993). Thereafter, several rice varieties have been transformed with genes encoding various Cry1A type Bt crystal proteins and have been shown to be resistant to one or more lepidopteran insect pests of rice (Nayak et al. 1997; Ye et al. 2003; Kim et al. 2009; Wang et al. 2014). First field trial of Bt rice was conducted in China in 1998 (Shu et al. 2000) and in 2009 China’s Ministry of Agriculture issued biosafety certificates for limited commercialization trial in Hubei Province in China for a 5-year period, 2009–2014 (Chen et al. 2011).

The large-scale deployment of transgenic crops expressing a single Bt toxin may lead to break-down of resistance in the field. To delay the resistance breakdown, the concept of gene pyramiding is advocated as one of the resistance management strategies. The *cry2A* genes are appropriate candidate for pyramiding with *cry1A* type gene due to variation in their structure and function. Use of synthetic genes expressing hybrid Bt toxins with increased potency is a promising strategy in genetic engineering.

A chimeric Bt gene, *cry2AX1* with part of sequence from *cry2Aa* and *cry2Ac* was developed in our laboratory to improve the insecticidal activity against a lepidopteran pest, *Helicoverpa armigera*. The chimeric Cry2AX1 protein exhibited higher level of toxicity than their parental proteins, Cry2Aa and Cry2Ac (Udayasuriyan et al. 2010). The Cry2AX1 protein isolated from recombinant Bt strain was also found to be effective against rice leaffolder. This chimeric gene was codon optimized to improve expression in crop plant (NCBI Accession Number GQ332539.1).

Another concern to be addressed while expressing Bt genes is optimal level of expression for desirable level of protection of crop plants against the target insect pest. One of the promising approaches is to target proteins to specific subcellular sites/compartments of plant cells, such as the chloroplast (Staub et al. 2000). Previously, Jang et al. (1999) reported that the targeting of foreign gene products to plastids using the transit peptide sequence increased the protein product levels in transgenic rice plants.

In the present study, we developed transgenic rice plants with codon optimized synthetic *cry2AX1* gene (fused with rice chloroplast transit peptide sequence) using *Agrobacterium* mediated transformation. The insect bioassay results indicated that the transgenic plants expressing synthetic *cry2AX1* gene were resistant to rice leaffolder.

## Materials and methods

### Construction of plant expression vector and rice transformation

**Fig. 1:**
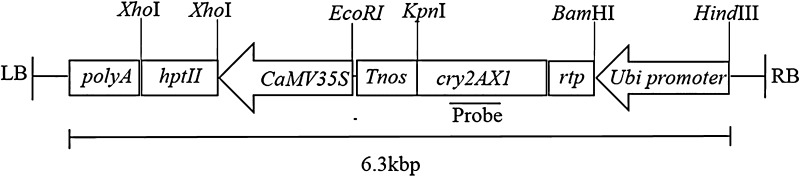
T-DNA region of plant transformation construct *pUH*-*rtp*-*2AX1.* Rice chloroplast transit peptide (*ctp*) was fused to *cry2AX1* gene. The *rtp*-*cry2AX1* gene is driven by a maize ubiquitin1 promoter and terminated by the *nopaline synthase* (*nos*) terminator. The plant selectable marker gene, *hptII* is under the control of the CaMV35S promoter and tailed by the CaMV35S polyA. *LB* and *RB* indicate left border and right border of T-DNA

**Fig. 2:**
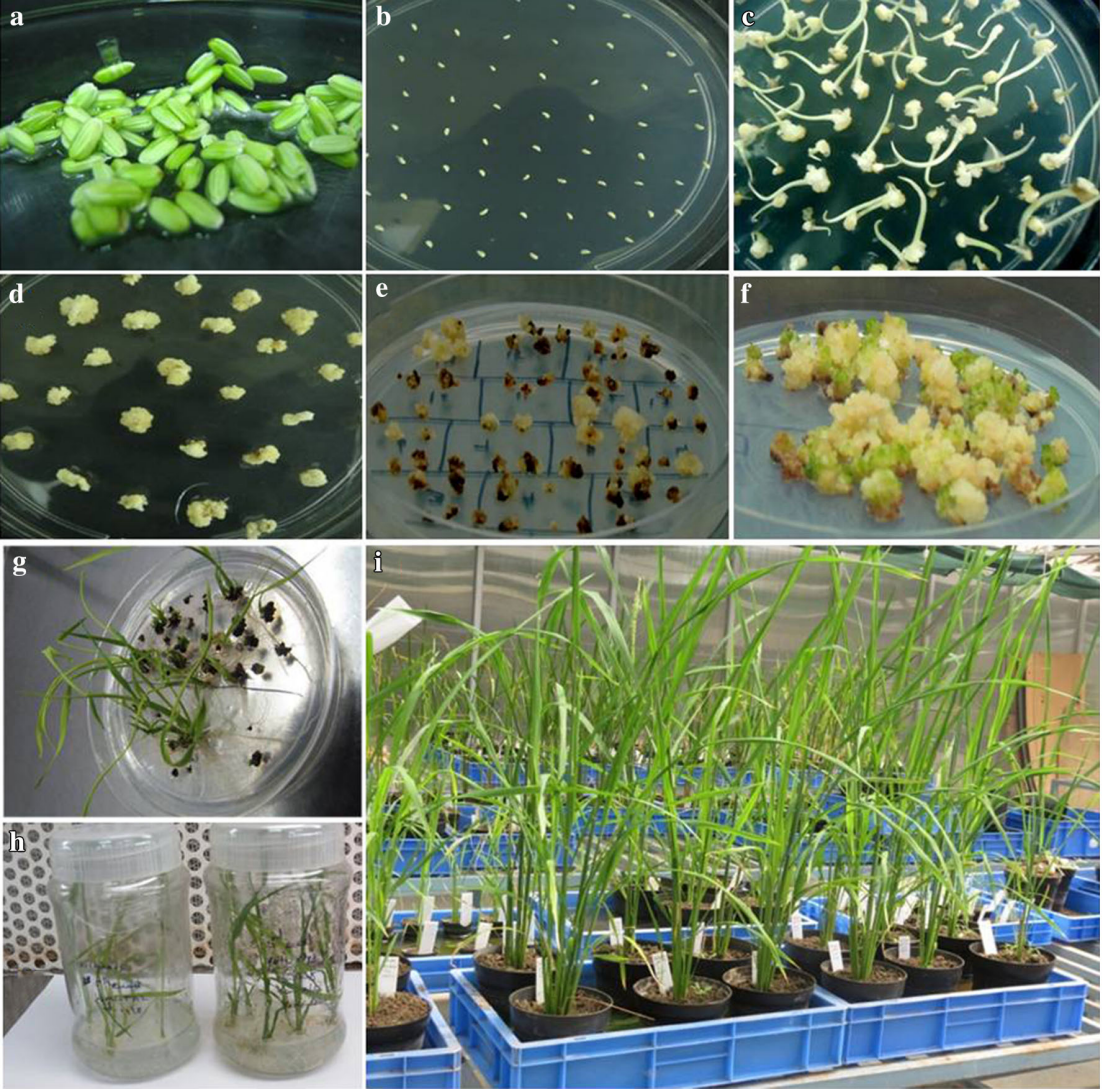
*Agrobacterium*-mediated transformation of rice (*Oryza sativa* L. cv. ASD16). **a** Immature seeds collected from rice; **b** pre-treated immature embryos infected with *Agrobacterium* on cocultivation medium; **c** callus initiation (and shoot tips) from co-cultivated embryo; **d** sub-cultured calli on resting medium; **e** callus proliferation on selection medium; **f** embryogenic calli on pre-regeneration medium; **g** regenerated transgenic rice plants; **h** transgenic rice plants in rooting medium; **i** established transgenic rice plants in transgenic greenhouse

The chimeric *cry2AX1* gene was constructed using DNA sequences corresponding to the 585 N-terminal and 48 C-terminal amino acids of Cry2Aa and Cry2Ac, respectively, which were isolated from indigenous strains of Bt. The amino acid sequence of Cry2AX1 differs from that of Cry2Aa for ten residues in a stretch of 24 amino acids (595–618) (Udayasuriyan et al. 2010). Codon optimised, synthetic *cry2AX1* gene (Accession Number GQ332539.1) was translationally fused at its 5′ end to the rice chloroplast transit peptide (rtp) sequence of *rbcS* gene (DNA of 148 bp corresponding to the rice *rbcS* transit peptide (rtp; Jang et al. 1999). The fusion gene driven by maize ubiquitin constitutive promoter was cloned in pUH binary vector (Agarwal et al. 2002) which harbours *hptII* (coding for *hygromycin phosphotransferase*), a plant selectable marker gene. The above construct, *pUH*-*rtp*-*2AX1* (Fig. 1) was mobilized into disarmed *Agrobacterium* strain, LBA4404 for rice transformation experiments. A popular rice variety, ASD16 was used for transformation. The *Agrobacterium*-mediated transformation was carried out according to the protocol described by Hiei and Komari (2008). Regenerated putative transgenic plants were maintained in transgenic greenhouse (Fig. 2).

### PCR and Southern blot hybridization analyses

PCR analysis was performed to demonstrate the presence of *cry2AX1* and *hptII* genes in putative transgenic lines of ASD16 using gene specific primers in T_0_ and T_1_ transgenic rice plants (Table 1). These primers amplify 800 and 630 bp internal fragments of *cry2AX1* and *hptII* genes, respectively. The plasmid DNA (pUH-*rtp*-*2AX1*) was used as positive control and DNA isolated from non-transformed control plants and the reaction mix without template DNA were used as negative control. The amplified PCR products were resolved on 1.2 % agarose gel, visualized on UV transilluminator upon ethidium bromide staining.

**Table 1:** List of primers used for polymerase chain reaction in the study

Primer name	Nucleotide sequence	Amplicon size
S2XSF2	CCTAACATTGGTGGACTTCCAG	~800 bp internal sequence of *cry2AX1* gene
S2XSR2	GAGAAACGAGCTCCGTTATCGT	
HF1	GCTGTTATGCGGCCATTGGTC	~630 bp internal sequence of *hptII* gene
HR1	GACGTCTGTCGAGAAGTTTG	
ActF1	GAGCGTTTCCGCTGCCCTGA	~500 bp internal sequence of Actin gene
ActR1	AGAAACAAGCAGGAGGACGGC	

Total genomic DNA was extracted from young leaf tissues of transformed plants of both T_0_ and T_1_ generation and non-transformed plants using the method described by Dellaporta et al. (1983). For Southern blot analysis, five µg of genomic DNA was digested overnight with *Hin*dIII, which has a recognition sequence at one end of the T-DNA. The digested DNA were separated on a 0.8 % agarose gel, and then transferred to a positively charged nylon membrane using 20X saline-sodium citrate (SSC) following standard capillary transfer protocol. The transferred DNA was cross-linked by a UV crosslinker at 1200 μJ min^−1^ for 1 min. The prehybridisation was carried out for 1 h and hybridization for 18 h at 60 °C. For hybridization, PCR amplified 800 bp internal region of *cry2AX1* gene was used as a probe. The probe DNA was labelled with α^32^P dCTP using Decalabel DNA labelling kit (Thermo Scientific Inc) and added to hybridization solution. After hybridization, the blot was washed with 3X SSC + 0.1 % SDS and 2X SSC + 0.1 % SDS for 15 min each, followed by 10 min in 0.5X SSC + 0.1 % SDS. All washings were carried out at 60 °C and the blot was exposed to X-ray film.

### RT-PCR analysis

Total RNA was isolated from PCR positive T_1_ plants along with a control plant using SV Total RNA Isolation System (Promega, USA) following manufacturer’s instructions. The first strand cDNA was synthesized using RevertAid™ First Strand cDNA Synthesis Kit (MBI Fementas, UK). The primers used for *cry2AX1* gene were same as used in PCR assay. The ActF1 and ActR1 primers were used for amplification of actin gene which was used as internal control (Table 1).

### Expression of Cry2AX1 protein in transgenic rice plants

The PCR positive T_0_ transgenic rice plants were subjected for quantitative ELISA at 30 DAS. In T_1_ progenies the concentration of Cry2AX1 protein was measured at three different phenological stages *viz*. vegetative (25 DAS), tillering (55 DAS) and reproductive (85 DAS) stages, for studying the temporal variation of *cry2AX1* gene expression. About 30 mg of tissues from top of the primary tiller in a plant was used for analysis. The tissue were homogenized in 500 µl of extraction buffer, spun at 3000*g* at 4 °C for 10 min and 100 µl of the supernatant was immediately used for assay. Each sample was replicated twice. Cry2AX1 protein expression in transgenic rice plants was determined by ELISA kit (Envirologix, USA) following standard procedures. The protein concentration was calculated on a linear standard curve, using the standards provided in the kit.

### Detached leaf bit bioassay

Bioassay studies for leaffolder resistance was conducted on ELISA positive T_0_ and T_1_ transgenic rice plants to study efficacy of the Cry2AX1 protein. Adults of rice leaffolder were collected from the rice field at Paddy Breeding Station, TNAU, Coimbatore and they were released on susceptible TN1 rice plants maintained in insect cages (65 cm × 65 cm × 75 cm) for culturing. After two generations, the neonate larvae of *C. medinalis* were used for the bioassay. Leaves of transgenic plants (35 days old seedling) were cut into pieces (about 3 cm length) and three leaf bits were placed on a moist filter paper in a plastic petriplate. Ten neonate larvae of rice leaffolder were released in each petriplate. A control was maintained using leaf bits collected from non-transgenic rice ASD16. Three replications were maintained and the experiment was carried out at 25 °C ± 1, 60 % relative humidity. Larval growth and mortality was recorded every day up to 5 days. After 5 days, leaf area damage and surviving larval characteristics were evaluated in transgenic as well as control plants. Percentage of leaf area damage was calculated using the formula:

$${\text{Leaf area damage }}\left( \% \right) = \frac{\text{Consumed leaf area}}{\text{Total leaf area}} \times 100.$$

### Statistical analysis

Values for concentration of Cry2AX1 protein and mortality of rice leaffolder are reported as mean ± SD. The association between protein expression and insect mortality was studied by correlation analysis using the Statistical Package for Social Studies (SPSS) software version 16.

## Results

### Molecular and biochemical analyses of putative T_0_ transgenic plants

**Fig. 3:**
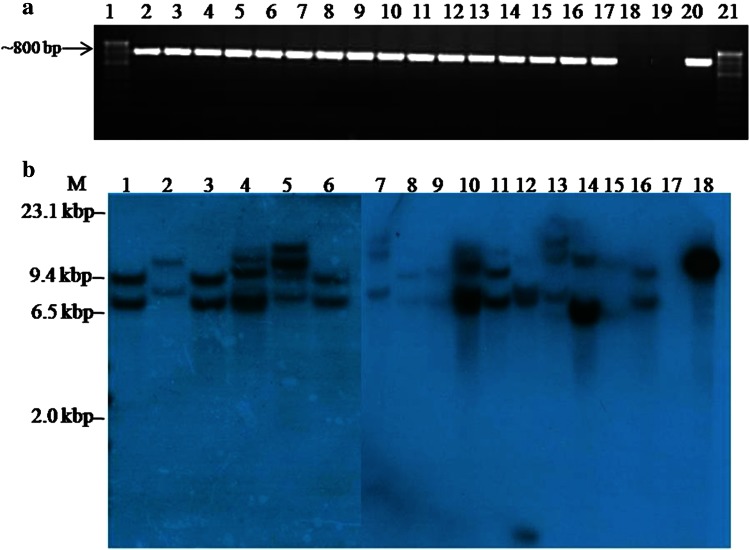
Molecular analysis of T_0_ Transgenic rice plants. **a** A 800 bp internal sequence of *cry2AX1* gene was amplified by PCR from the DNA isolated from putative transgenic plants. *Lanes* 1 and 21, 100 bp marker; *Lanes* 2–17, Putative transgenic plants of ASD16; *Lane* 18, Non transformed control plant; *Lane* 19, Negative control (water); *Lane* 20, *pUH*-*rtp*-*2AX1* plasmid as a positive control. **b** Southern blot hybridization analysis of T_0_ transgenic rice plants expressing *cry2AX1* gene. DNA sample isolated from transgenic and non-transgenic plants digested with *Hin*dIII restriction enzyme, DNA fragments separated by electrophoresis, transferred to nylon membrane and allowed to hybridize with a radioactively labelled 800 bp internal sequence of *cry2AX1* gene. *M*, λ/*Hin*dIII marker; *Lanes* 1–16, Genomic DNA from transgenic rice plants UR1, UR2, UR3, UR4, UR5 UR6, UR7, UR8, UR9, UR10, UR11, UR12, UR13, UR14, UR15, UR16, respectively; *Lane* 17, Non-transformed control plant; *Lane* 18, Positive control (*pUH*-*rtp*-*2AX1*)

Twenty putative transgenic rice lines were regenerated and putative transgenic plants were transferred to greenhouse for molecular analyses (Fig. 2). Total genomic DNA from putative rice transformants was subjected to PCR analysis with *cry2AX1* and *hptII* gene specific primers. Out of 20 plants regenerated, 16 were found to be positive for the amplification of ~800 bp (Fig. 3a) and 630 bp internal sequences of *cry2AX1* and *hptII* gene, respectively. Southern hybridization analysis showed two hybridization signals (range 6.7–12.0 kbp) in eight of the 16 plants analyzed whereas the remaining eight plants had three to five signals (range 6.5–19.0 kbp). The non-transformed control plant did not show any hybridization signals (Fig. 3b).

The PCR positive transgenic rice lines were further screened by quantitative ELISA kit. All the 16 PCR positive plants were found positive for the expression of Cry2AX1 protein. The expression of Cry2AX1 protein in these transgenic rice lines ranged 10.40–120.50 ng/g fresh leaf tissue (Table 2). One of the T_0_ plants, UR11 recorded maximum level of expression of Cry2AX1 protein i.e., 120.50 ng/g fresh leaf weight.

**Table 2:** Quantitative ELISA and rice leaffolder bioassay on T_0_ rice lines expressing *cry2AX1* gene

Rice line	Cry2AX1 concentration in fresh leaf tissue^a^ Mean ± SD	Mortality of *C. medinalis* ^b^ (%)
UR1	22.45 ± 0.85	30.00 ± 0.00
UR2	30.80 ± 0.80	30.00 ± 0.00
UR3	38.30 ± 1.70	30.00 ± 0.00
UR4	68.00 ± 2.00	50.00 ± 0.00
UR5	23.30 ± 0.00	30.00 ± 0.00
UR6	22.45 ± 0.85	NT
UR7	24.15 ± 0.85	23.33 ± 5.77
UR8	12.45 ± 0.85	NT
UR9	35.00 ± 0.00	43.33 ± 5.77
UR10	94.00 ± 1.00	50.00 ± 0.00
UR11	120.50 ± 0.50	80.00 ± 0.00
UR12	29.50 ± 0.50	33.33 ± 5.77
UR13	38.00 ± 0.00	43.33 ± 5.77
UR14	20.80 ± 1.60	NT
UR15	14.40 ± 0.00	NT
UR16	10.40 ± 0.80	NT
Control	0	0.0

*NT* not tested, *SD* standard deviation

^a^Mean of two replications

^b^Mean of three replications

### Inheritance studies

**Fig. 4:**
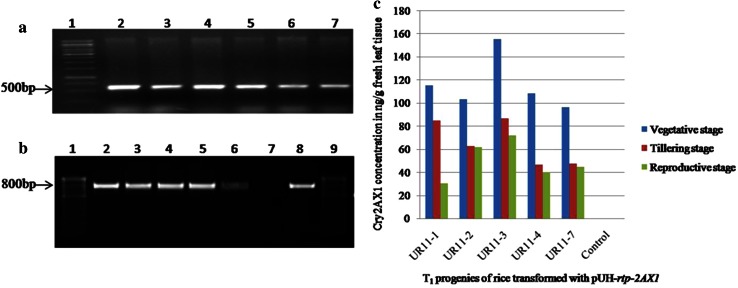
**a** RT-PCR analysis of rice actin (housekeeping gene) in T_1_ transgenic plants. *Lanes* 1, 100 bp ladder; *Lanes* 2–6, transgenic plants (UR11–1, UR11–2, UR11–3, UR11–4 and UR11–7); *Lane* 7, non-transgenic plants (Wild type ASD16). **b** RT-PCR analysis of *cry2AX1* in T_1_ transgenic plants. *Lane* 1 and 9, 100 bp ladder; *Lanes* 2–6, transgenic plants (UR11–1, UR11–2, UR11–3, UR11–4 and UR11–7); *Lane* 7, non transgenic plants (Wild type ASD16), *Lane* 8, Positive control (*pUH-rtp-2AX1*). **c** The temporal expression of Cry2AX1 protein in T_1_ transgenic rice plants. The Cry2AX1 protein concentration in fresh leaf of T_1_ transgenic plants at vegetative, tillering and reproductive stage

**Fig. 5:**
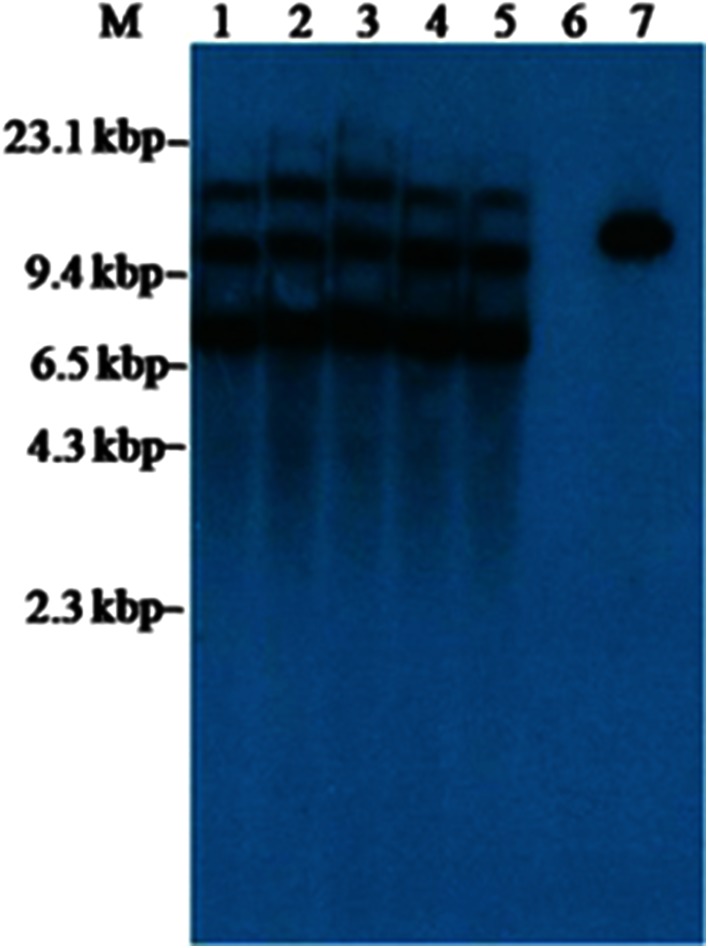
Southern blot hybridization analysis of T_1_ transgenic rice plants expressing *cry2AX1* gene. DNA digested with *Hin*dIII and probed with a radioactively labelled 800 bp internal sequence of *cry2AX1* gene. *M*, λ/*Hin*dIII marker; *Lane* 1, UR11–1; *Lane* 2, UR11–2; *Lane* 3, UR11–3; *Lane* 4, UR11–4; *Lane* 5, UR11–7; *Lane* 6, Control plant; *Lane* 7, Positive control (*pUH*-*rtp*-*2AX1*)

Thirty plants of T_1_ progenies derived from transgenic rice line UR11 (selected based on relative levels of Cry2AX1 protein in T_0_ generation) were established in the greenhouse for further studies on gene inheritance, expression and efficacy. An amplicon of ~800 bp was found in all the 30 T_1_ progenies for the presence of *cry2AX1* gene, whereas no amplification was found in non-transgenic control plant. RT-PCR analysis on a randomly selected five T_1_ plants showed presence of transcripts of the *cry2AX1* (Fig. 4b) and actin gene (Fig. 4a). Further, expression of Cry2AX1 protein in these plants was analyzed at three phenological stages *viz*, vegetative, tillering and reproductive and the concentration of Cry2AX1 protein varied from 96.00 to 155.00, 46.40 to 86.40 and 30.40 to 72.00 ng/g fresh leaf tissue, respectively (Fig. 4c). The Cry2AX1 protein level in seeds ranged 3.2–4.8 ng/g fresh seed (Supplementary Table 1). Genomic DNA from five ELISA positive T_1_ progeny plants of UR11 was digested by *Hin*dIII enzyme and subjected to Southern blot hybridization using *cry2AX1* probe. All the five T_1_ progenies (UR11–1, UR11–2, UR11–3, UR11–4 and UR11–7) showed three hybridization signals of ~7.5, ~10.0 and ~14.5 kbp (Fig. 5).

### Detached leaf bit bioassay

In order to determine the insecticidal activity of Cry2AX1 protein expressed in transgenic rice plants, detached leaf bit bioassay was carried out using neonate larvae of rice leaffolder on the ELISA positive T_0_ plants. The larval mortality ranged 23.33–80.00 % (Table 2). A significant, positive correlation was found between the concentration of Cry2AX1 and insect mortality in T_0_ transgenic plants (*r* = 0.920, *p* = 0.01). No larval mortality was observed on control plants and the major portion of the leaf tissue was consumed by the surviving larvae over a period of 5 days (Supplementary Figure 1).

**Fig. 6:**
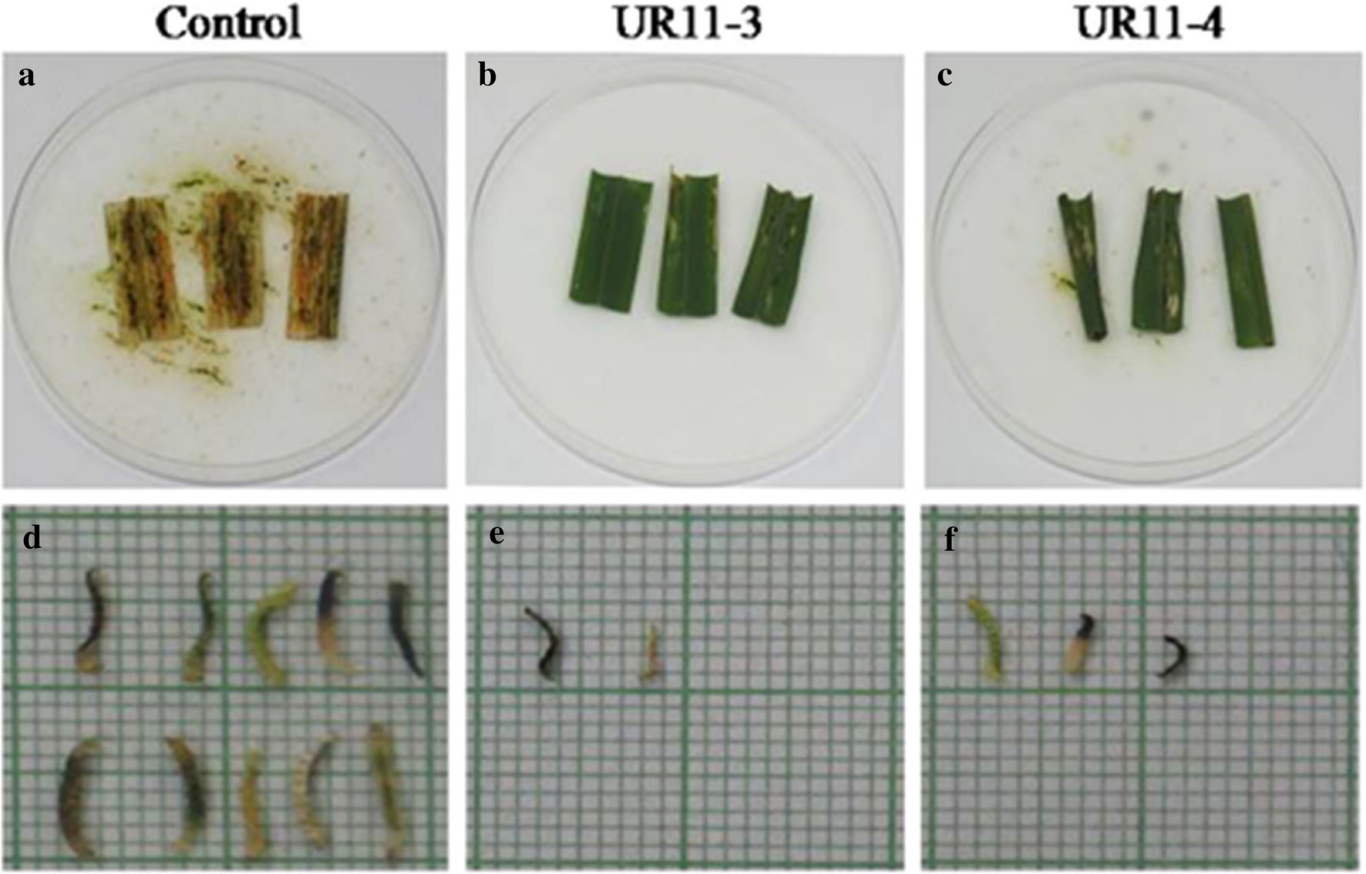
Detached leaf bit bioassay against rice leaffolder (*C. medinalis*) in T_1_ transgenic rice plants expressing Cry2AX1 protein. **a** Non transformed control plant (ASD16); **b** and **c** transformed rice plant (UR11–3 and UR11–4); **d**, **e** and **f** size of survivor from non transformed and transformed rice plants

The efficiency of T_1_ transgenic plants (Three T_1_ progeny plants) expressing Cry2AX1 protein (which expressed relatively higher levels of Cry2AX1 protein) with respective controls were tested against the neonates of rice leaffolder. Mortality in rice leaffolder varied from 53.33 to 80.00 % in the T_1_ transgenic rice plants (Table 3). The percentage of leaf area damaged by rice leaffolder larvae varied from 20.29 to 36.32 % in the T_1_ transgenic lines compared to 92.73 % in control plants (Fig. 6).

**Table 3:** Detached leaf bit bioassay on T_1_ transgenic rice plants expressing *cry2AX1* gene

S. no.	Progeny no.	*C. medinalis* mortality^a^ (%) (56 DAS) Mean ± SD	Leaf area damage^a^ (%)
1	UR11–1	63.33 ± 4.71	32.26 ± 0.64
3	UR11–3	80.00 ± 0.00	20.29 ± 1.06
4	UR11–4	53.33 ± 4.71	36.32 ± 0.85
6	Control	0	92.73 ± 0.43

^a^Mean of three replicates

## Discussion

Transgenic plants with insecticidal crystal protein from *Bacillus thuringiensis* could drastically reduce the use of broad-spectrum insecticides against insect pests. However, there is a risk that insect could become resistant to the Bt toxin. Therefore, it is envisaged that gene pyramiding in transgenic plants could be apossible strategy for simultaneous expression of two or more *cry* genes (Datta et al. 2002). To improve the insecticidal property of the Cry toxin, a chimeric Bt gene (*cry2AX1*) was developed with *cry2Aa* and *cry2Ac* sequences from indigenous isolates of Bt. Our earlier study proved that the chimeric Cry2AX1 protein expressed in Bt was highly toxic to the larvae of the rice leaffolder, *C. medinalis* (Manikandan et al. 2014).

A number of studies have suggested that chloroplasts are ideal sites for the expression of transgenes (Jang et al. 1999; Kim et al. 2009; Verma and Daniell 2007). Earlier studies reported improved levels of expression of foreign genes by targeting them to the chloroplast in transgenic tobacco, cotton and rice (Jang et al. 1999; Kim et al. 2009; Rawat et al. 2011). In the present study, we introduced a codon optimised synthetic *cry2AX1* gene driven by ubiquitin promoter with rice chloroplast transit peptide for targeting Cry2AX1 protein into the chloroplast.

In this study, twenty putative transformants of rice were generated on hygromycin selection, of which 16 plants were found to be positive for the *cry2AX1* gene in PCR assay. The presence of transgene was confirmed by expression analysis of 16 PCR positive T_0_ transgenic plants by ELISA. All the 16 primary transformants gave positive results for the expression of Cry2AX1 protein and concentration of Cry2AX1 protein in putative transformants ranged 10.40–120.50 ng/g of leaf tissue on fresh weight. Similar variation in expression level was reported by earlier researcher on Bt rice (Wu et al. 2001). Genetic background and gene constructs were shown to influence the level of expression (Maqbool et al. 2001; Breitler et al. 2000; Meiyalaghan et al. 2006). Plant to plant variation in expression is mainly due to integration of transgenic DNA into regions of the genome that are transcriptionally repressed (heterochromatin), which ultimately leads to transgene silencing. These types of position effects often result in the production of transgenic lines exhibiting high and low levels of expression (De Bolle et al. 2003).

Stable integration of transgene was confirmed by Southern hybridization analysis and Southern results indicated integration of *cry2AX1* gene at two to five locations in the rice genome. In most of the cases, integration of *cry2AX1* gene was at two loci (a relatively simple pattern) and in a few cases, it was 3–5 (complex pattern). Several earlier researchers reported that single copy transgenes had exhibited higher expression levels than multiple T-DNA insertions (Hobbs et al.^1990^; Jones et al. 1987). However, we observed that level of expression of transgene and resistance was higher in the multiple copy line UR11 compared to other transgenic lines. Zaidi et al. (2009) also reported that the level of Cry1C expression was higher in transgenic rice plant with three copies of *cry1C* gene when compared to single copy plants.

Detached leaf bit bioassay of the selected T_0_ plants against neonate larvae of rice leaffolder revealed significant variability in larval mortality. The mortality varied from 23.33 to 80.00 % among the T_0_ transformants. The effect of the Cry2AX1 protein was also seen on the larva as there was considerable differences in the size of the larva that fed on the transgenic and wild type. Correlation between the Cry2AX1 protein expression and larval mortality showed a highly significant positive relationship (*r* = 0.920) indicating that higher the concentration of Cry2AX1 protein has resulted in higher mortality of the larvae. Transformants expressing higher levels of Cry2AX1 protein invariably induced higher larval mortality.

The T_1_ progenies of one of the primary transformants (UR11) were subjected to PCR analysis to determine the stable inheritance of transgene. The Mendalian segregation ratio could not be determined due to smaller size (30) of the T_1_ progeny. Southern hybridization analysis revealed three integrations of *cry2AX1* gene in five of the T_1_ progenies tested at random (event UR11).

Temporal variation of Cry2AX1 protein was quantified in T_1_ transgenic rice plants (Five T_1_ progeny plants) at three phenological stages (vegetative, tillering and reproductive). The results showed a decline in expression of Cry2AX1 when the crop progressed towards the maturity. The level of expression was relatively higher at vegetative stage when compared to other growth periods. One of the progenies i.e., UR11–3 showed the highest level of expression 155.00 ng/g of fresh leaf weight and the transgenic seeds showed significantly low level of expression varying from 3.2 to 4.8 ng/g fresh seed. The ELISA results indicated that the expression of Cry2AX1 protein in leaves decreased towards the end of growing season. Similar results were also reported in previous researches on Bt rice (Wu et al. 2001; Zhao et al. 2004; Han et al. 2009). Wan et al. (2005) reported that expression pattern of Bt toxin in progenies of cotton varies within the season with higher concentration at the beginning and lower at the latter stages. The possible reason for reduction of Bt protein expression is that the level of *cry* gene transcripts decreased in plant tissues as they mature and is likely because of decreased activity of the promoters, which is probably hindered by adverse physiological conditions in the rice plants (Christensen et al. 1992). Bakhsh et al. (2012) reported that the reduction of Bt protein content in later season of cotton tissue could be attributed to the over expression of the Bt gene at earlier stage, which leads to gene regulation at post-transcriptional level and consequently results in gene silencing at a later stage.

Insect bioassay on T_1_ transgenic rice plants resulted in larval mortality ranging from 53.33 to 80.00 % with a low level of leaf damage (20.29–36.32 %) whereas in control plant there was no mortality and major portion of leaf tissue (92.73 %) was consumed by surviving larvae over the period of 5 days. The larvae recovered from control plants were well-developed and progressed to next instar (third instar), while the larvae recovered from those of transgenic plants were dead or sluggish, weak and underdeveloped. The reduction in the larval size was evident among the larvae tested on the transgenic rice in the bioassay. This may be because of low level of feeding, which in turn resulted in reduced accumulation of toxins in insects that did not cause the mortality but brought about the reduction in size.

It has been demonstrated by earlier researchers that the Cry protein concentration is directly related to the level of insect-resistance. Chen et al. (2005) reported an insect mortality of 100 % against rice stem borer in plants with average level of Cry2A protein expression of 10 µg/g fresh weight. Riaz et al. (2006) reported a concentration of Cry1Ac protein ranging between 4.6 and 16 µg/g and Cry2A at a level of 0.34–1.45 µg/g of tissue in leaves of transgenic rice plants. In our studies, the generated *cry2AX1* transgenic rice plants showed low level of Cry2AX1 expression. Therefore, the level of expression of Cry protein reported by earlier workers indicate that a relatively higher level of *cry2AX1* gene expression may be necessary for a desirable level of insecticidal activity in rice plants and this can be achieved by generating and screening more number of putative transformants.

## Electronic supplementary material

Below is the link to the electronic supplementary material.

Supplementary material 1 (DOCX 736 kb)
