# Supplementary material for: Transgenic rice plants expressing synthetic cry2AX1 gene exhibits resistance to rice leaffolder (Cnaphalocrosis medinalis)
Source: 3 Biotech. 2016 Jan 5;6(1):10. doi: 10.1007/s13205-015-0315-4 (PMC4701705; doi:10.1007/s13205-015-0315-4)
Supplement: Supplementary file 1 — Supplementary material 1 (DOCX 736 kb) [file 13205_2015_315_MOESM1_ESM.docx]

**Supplementary table**

**Table 1 Expression of Cry2AX1 protein in T_1_ progenies of rice**

| **S. No** | **Transgenic (T_0_) line** | **T_1_**  **Progeny** | **Cry2AX1 concentration in fresh leaf tissue ( ng/g )***  **Mean ± SD** | | | |
| --- | --- | --- | --- | --- | --- | --- |
|  |  |  | **Vegetative stage**  **(25 DAS)** | **Tillering stage**  **(55 DAS)** | **Reproductive stage**  **(85 DAS)** | |
|  |  |  |  |  | **Leaves** | **Seed** |
| 1 | UR 11 | UR11-1 | 115.00±0.50 | 84.80±0.00 | 30.40±0.00 | 3.2±0.00 |
| 2 |  | UR11-2 | 103.00±0.00 | 62.40±0.00 | 61.60±0.80 | NT |
| 3 |  | UR11-3 | 155.00±0.00 | 86.40±0.00 | 72.00±0.00 | 4.8±0.00 |
| 4 |  | UR11-4 | 108.00±0.00 | 46.40±0.00 | 40.00±0.00 | NT |
| 5 |  | UR11-7 | 96.00±0.00 | 47.20±0.80 | 44.80±0.00 | 4.2±0.00 |
| 6 | Control | | 0 | 0 | 0 | 0 |

*Mean of two replicates

**Supplementary figure**


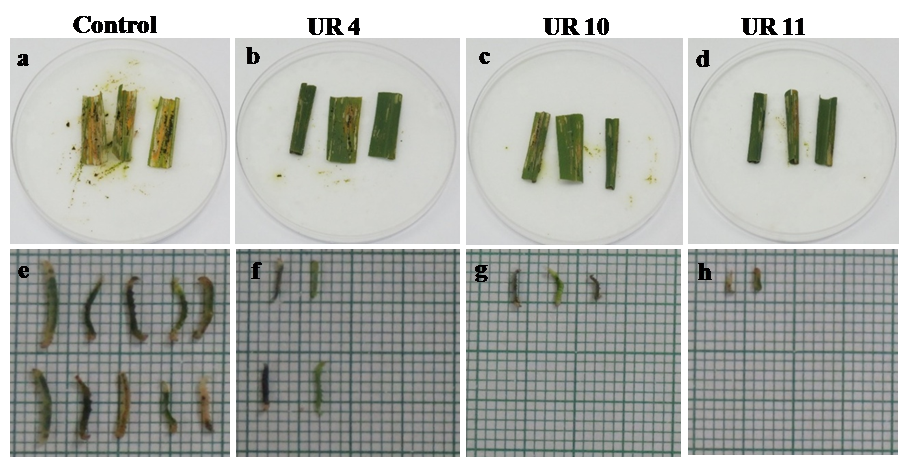


**Fig. 1 Detached leaf bit bioassay against rice leaf folder (*C. medinalis*) in T_0_ transgenic rice plants expressing Cry2AX1 protein.**

**a** non transformed control plant (ASD16); **b**, **c** and **d** transformed rice plant (UR4, UR10 and UR11); **e**, **f**, **g** and **h** size of survivor from non transformed and transformed rice plants.
